# Suppression and Regression of Choroidal Neovascularization in Mice by a Novel CCR2 Antagonist, INCB3344

**DOI:** 10.1371/journal.pone.0028933

**Published:** 2011-12-19

**Authors:** Ping Xie, Motohiro Kamei, Mihoko Suzuki, Nagakazu Matsumura, Kentaro Nishida, Susumu Sakimoto, Hirokazu Sakaguchi, Kohji Nishida

**Affiliations:** Department of Ophthalmology, Graduate School of Medicine, Osaka University, Suita, Osaka, Japan; University of Leuven, Rega Institute, Belgium

## Abstract

**Purpose:**

To investigate the effect of an intravitreally administered CCR2 antagonist, INCB3344, on a mouse model of choroidal neovascularization (CNV).

**Methods:**

CNV was induced by laser photocoagulation on Day 0 in wild type mice. INCB3344 or vehicle was administered intravitreally immediately after laser application. On Day 14, CNV areas were measured on retinal pigment epithelium (RPE)-choroid flat mounts and histopathologic examination was performed on 7 µm-thick sections. Macrophage infiltration was evaluated by immunohistochemistry on RPE-choroid flat mounts and quantified by flow cytometry on Day 3. Expression of vascular endothelial growth factor (VEGF) protein in RPE-choroid tissue was examined by immunohistochemistry and ELISA, VEGF mRNA in sorted macrophages in RPE-choroid tissue was examine by real-time PCR and expression of phosphorylated extracellular signal-regulated kinase (p-ERK 1/2) in RPE-choroid tissue was measured by Western blot analysis on Day 3. We also evaluated the efficacy of intravitreal INCB3344 to spontaneous CNV detected in Cu, Zn-superoxide dismutase (SOD1) deficient mice. Changes in CNV size were assessed between pre- and 1week post-INCB3344 or vehicle administration in fundus photography and fluorescence angiography (FA).

**Results:**

The mean CNV area in INCB3344-treated mice decreased by 42.4% compared with the vehicle-treated control mice (p<0.001). INCB3344 treatment significantly inhibited macrophage infiltration into the laser-irradiated area (p<0.001), and suppressed the expression of VEGF protein (p = 0.012), VEGF mRNA in infiltrating macrophages (p<0.001) and the phosphorylation of ERK1/2 (p<0.001). The area of spontaneous CNV in *Sod1* ^−/−^ mice regressed by 70.35% in INCB3344-treated animals while no change was detected in vehicle-treated control mice (p<0.001).

**Conclusions:**

INCB3344 both inhibits newly forming CNV and regresses established CNV. Controlling inflammation by suppressing macrophage infiltration and angiogenic ability via the CCR-2/MCP-1 signal may be a useful therapeutic strategy for treating CNV associated with age-related macular degeneration.

## Introduction

Age-related macular degeneration (AMD) is the leading cause of legal blindness among elderly people in developed countries [1]. The majority of these patients with severe vision loss have retinal damage by choroidal neovascularization (CNV) [2], which is the hallmark of wet AMD and is defined as new blood vessels arising from choriocapillaris and extending into the sub-retinal pigment epithelium (sub-RPE) [3], sub-retinal space, or both. Although the pathogenesis of CNV is not completely understood, inflammatory processes, especially the infiltration of inflammatory cells, are recognized as an important mediator of CNV formation [4].

Macrophage accumulation in the CNV area and expression of angiogenic cytokines, such as vascular endothelial growth factor (VEGF) are involved in CNV formation [5]–[10]. Moreover, in mice depleted of macrophages, CNV is reduced and VEGF production is decreased [11], [12], which suggests a role for macrophages as producers and regulators of angiogenic factors in the pathogenesis of CNV.

The chemokine receptor CCR2 and its ligand, monocyte chemoattractant protein-1 (MCP-1; also known as CCL2) represent a critical signaling pathway responsible for the recruitment of monocyte-macrophages [13]–[16]. MCP-1 expression is not detectable [2], [17] or very low [18] in healthy young adult mice, but is found in high concentrations in the eyes of CNV bearing mice with the infiltration of macrophages [2], [17], [19], [20]. Recent studies also demonstrate an association between elevated urinary MCP-1 level and AMD [21], and the intraocular elevation of MCP-1 in AMD patients with CNV [22]. Mice deficient in CCR2 or MCP-1, have a marked impairment in macrophage influx into sites of inflammation [23]–[25] and are protected from inflammatory diseases in a range of animal models [26]–[29]. The reduction in the number of infiltrating macrophages and the following amelioration of CNV formation also occurs in CCR2 KO mice [16] or MCP-1 KO mice [30]. Therefore, the inhibition of macrophages by blockage of the CCR2/MCP-1 signal has emerged as a novel therapeutic target for CNV treatment.

Recently, INCB3344, a potent and selective small molecule antagonist of CCR2, was discovered and was demonstrated to have a high ability to compete with MCP-1 [26]. INCB3344 rapidly binds both rodent [26] and human CCR2 [27] with a high affinity, inhibits CCR2 binding to MCP-1, and blocks MCP-1-induced signaling and functioning in CCR2-expressing cells. Blocking the CCR2/MCP-1 signal by INCB3344 suppresses macrophage recruitment and attenuates the signs and symptoms of a variety of inflammatory diseases such as peritonitis, delayed-type hypersensitivity, experimental autoimmune encephalomyelitis, atherosclerosis, arthritis and thermal hyperalgesia in animal models [26], [28], [29]. All of these lines of evidence suggest that INCB3344 acts as an effective and ideal tool for treating inflammatory diseases. Given the close relationship between inflammation and neovascularization, we hypothesized that INCB3344 might be of therapeutic value in treating CNV. In this study, we administered INCB3344 to mouse models with different phases of CNV to determine whether this compound has the ability to suppress and regress CNV. We also investigated the possible molecular mechanism of INCB3344 involved in CNV formation.

## Materials and Methods

### Animals

Male wild-type C57BL/6 mice (Charles River, Japan) 8 weeks of age were used as the laser induced CNV mouse model. Cu, Zn-superoxide dismutase (SOD1)-deficient mice with a C57BL/6 background (Jackson Laboratory, U.S.A.) were generated as described [24] and used as an established CNV model. Anesthesia was induced by peritoneal injection of 50 mg/kg ketamine HCl (Sankyo, Tokyo, Japan) and 10 mg/kg xylazine (Bayer, Tokyo, Japan), and the pupils were dilated with topical 1% tropicamide (Santen, Osaka, Japan). The animals were cared for in accordance with the Association for Research in Vision and Ophthalmology (ARVO) Statement for the Use of Animals in Ophthalmic and Vision Research. All animal experiments were carried out in accordance with a protocol approved by the Institutional Animal Care and Use Committee of Osaka University (#20–094-0).

### Laser induced CNV and drug treatment

Laser photocoagulation (514 nm Argon laser, 130 mW, 100 ms duration, 75 µm spot size; Ultima 2000 SE, Lumenis/Coherent, Palo Alto, CA, USA) was performed bilaterally in each wild-type C57BL/6 mouse. Laser spots were applied in a standard fashion around the optic nerve using a slit lamp delivery system (Carl Zeiss, Germany) and using a handheld cover slip as a contact lens. Only burns that produced a bubble, indicating rupture of the Bruch membrane, were included in the study.

INCB3344 (PF-418725, MW577.6) was supplied by Pfizer (New York, USA) and shown to be safe to the mouse retina at concentrations of zero-1800 nM in previous toxicity experiments (Data not be shown here). Here, we selected the highest dose of INCB3344 (1800 nM) in the safe range in our experiment. Immediately after laser photocoagulation, mice were randomized into two groups and received intravitreal injections of 1 µl INCB3344 (1800 nM) or 1 µl vehicle (dimethyl sulfoxide dissolved in phosphate buffered saline, PBS). Intravitreal injection was performed with the FemtoJet Microinjector System (Eppendorf, Germany) under a high magnification stereomicroscope (Leica MS5, Germany).

### Histological examination

For histological examination, 3 mice in each group were sacrificed on Day 14 after the treatments. The eyes were enucleated and fixed with 4% PFA for 1 hour at 4°C. After removing the anterior segment, the eyecups were fixed again in 4% PFA overnight, dehydrated in 30% sucrose for 6 hours, and then embedded in Tissue-Tek® O.C.T. Compound (Sakura Finetek, Japan). The eyecups were sectioned into 7 µm-thick slices and stained with haematoxylin and eosin. Sections were examined using an Olympus BX50 microscope (Olympus, Japan), and images were digitalized using an Axiocam HRc camera and Axiovision version 3.1 image capture software (Carl Zeiss, Germany).

### Measurement of laser-induced CNV size

On Day 14 after laser photocoagulation, the sizes of CNV lesions were measured on RPE-choroid flat mounts by a similar method to that described previously [31]. In brief, mice were deeply anaesthetized and perfused with 1 ml of phosphate-buffered saline containing 50 mg/ml fluorescein-labelled dextran (#FD2000S-1G, Sigma, MO, USA). Then mice were sacrificed humanely, and the eyes were enucleated and fixed in 4% paraformaldehyde (PFA) for 1 hour. The anterior segment of the eye was cut off, and the vitreous and the entire retina were carefully removed from the eyecups. Four or five radial cuts in the remaining RPE-choroid-sclera were made from the edge to the equator, and the eyecups were flat-mounted in PermaFluor™ Aqueous Mounting Medium (Thermo, USA) with the retinal pigment epithelium (RPE) layer facing up. Those flat mounts were examined and recorded by the same microscope as before. 78 spots from 14 vehicle treated mice and 81 spots from 14 INCB3344 treated mice were examined, excluding eyes with hemorrhages. Image J for Windows (NIH, Bethesda, Maryland) analysis software was used to measure the area of CNV, with the operator blinded with respect to treatment groups.

### Immunohistochemistry of macrophages and VEGF

To investigate possible cellular and molecular responses to INCB3344 administration to the CNV model, we examined the status of macrophages and related angiogenic cytokines. After laser photocoagulation, 3 mice in each group were sacrificed and eyes were enucleated on Day 3, and 7 µm cryosections were prepared for immunohistochemistry. The protocols for preparing the cryosections here were almost the same as those mentioned previously, except that the fixation time was shortened to 6 hours. The cryosections were blocked with 5% bovine serum albumin (BSA) for 1 hour at room temperature. Primary antibodies against mouse F4/80 (1∶500, Catalog No. BM40075, Monoclonal Antibody to Mouse Macrophages: F4/80, Acris, Germany) and mouse VEGF (1∶200, Catalog No. ab46154, Rabbit polyclonal to VEGF, Abcam, USA) were incubated overnight at 4°C. Those slides omitting primary antibodies were used as negative control. After three washes in PBS, the slides were incubated with fluorescent–tagged secondary antibodies (Alexa-Fluor 488 and Alexa-Fluor 546; Invitrogen, Carlsbad, CA) and DAPI for 1 hour at room temperature. Sections were washed again and mounted in mounting medium and coverslipped.

Macrophages were also detected by F4/80 antibody in the choroid-RPE flat mounts on Day 3. All the procedures were performed at 4°C. In brief, 3 mice in each group were sacrificed and the eyes were enucleated and fixed with 4% paraformaldehyde for 30 minutes. Then washed three times in PBS, followed by dehydration and rehydration through a methanol series (40%, 80%, 100%, 80%, 40%). Washing again, eye cups were obtained by removing the anterior segments. After blocking with 5% BSA for 1 hour, eye cups were incubated 48 hours with F4/80 antibody. After five washings with PBS (1 hour each time), the eyecups were incubated with Alexa 546–tagged secondary antibodies and DAPI overnight. The eyecups were washed again and flat-mounted as previously mentioned. These slides were examined under a fluorescence microscope (AX80; Olympus, Tokyo, Japan).

### Flow cytometry of macrophages

In order to quantify the number of macrophages, eyes were enucleated on Day 3. The RPE-choroid complexes were separated and disrupted with a sharp micro-scissor into small pieces. Then they were treated with collagenase D (2 mg/mL, Roche, Germany) on a Bio-shaker at 37°C for 1 hour. After that, they were filtered, and the single-cell suspensions were incubated in Fc block (1∶100, Catalog No. 14-0161, eBiosciences, USA) for 15 minutes on ice, then stained with FITC-conjugated anti-mouse F4/80 (1∶30, Catalog No. 11-4801, eBiosciences, USA) or FITC-conjugated isotype (1∶100, Catalog No. 11-4321, eBiosciences, USA). Live cells were detected by gating on forward versus side scatter [4], followed by analysis of F4/80 in the fluorescence channel (FACSCalibur; BD Biosciences, USA). At least 50,000 viable cells were analyzed per condition. Data were analyzed by the system software (Cellquest software; BD Biosciences, USA). A total of 5 mice were examined per group. The number of ocular-infiltrating macrophages was calculated from the percent of each population in the gate of the precounted, total number of viable cells using trypan blue dye exclusion.

### Macrophage sorting and quantitative real-time PCR analysis (qPCR) of VEGF

For macrophage sorting, eyes were enucleated on Day 3. Single cells were isolated and stained as the described above. Cells showing FITC-F4/80 signals were collected by a FACSAria flow cytometer (BD Biosciences, USA) with FlowJo software (Tree Star. OR, USA).

Total RNA was extracted from sorted macrophages in each group using the RNeasy Plus Mini kit (Catalog No. 74134 Qiagen, Valencia, CA) and transcribed into cDNA using ExScript RT reagent kit (Takara Bio, Otsu, Shiga, Japan) according to the manufacturer's protocol. Real-time PCR analysis was performed by Platinum SYBR Green qPCR SuperMix-UDG (Catalog No. 11733-038, Invitrogen, Carlsbad, CA). The reaction was carried out for 40 cycles of 15 seconds at 95°C and 1 minute at 60°C after an initial incubation at 95°C for 10 minutes. The levels of PCR products were monitored with the Mx3000P QPCR System (Stratagene, USA). The baseline and threshold were adjusted according to the manufacturer's instructions. The relative abundance of transcripts was normalized using either the expression level of GAPDH mRNA or VEGF-A mRNA by ΔΔCt method. Three individual gene-specific values thus calculated were averaged. The primers used in this experiment are as follows- Mouse VEGF-A: sense 5′-AGCCGAGCTCATGGACGGGT-3′ and antisense 5′-AGTAGCTTCGCTGGTAGACATC-3′; Mouse GAPDH: sense 5′-TGGCAAAGTGGAGATTGTTGCC-3′ and antisense 5′-AAGATGGTGATGGGCTTCCCG-3′. At least 8 eyes were needed to obtain a sufficient number of enriched macrophages for qPCR analysis in the above process.

### Enzyme-linked immunosorbent assay (ELISA) of VEGF

To quantify VEGF protein levels, the RPE-choroid complexes were micro-surgically isolated from the eyes on Day 3, and placed immediately into 100 µl RIPA buffer (R0278, Sigma) supplemented with 1% Protease inhibitor cocktail (P8340, Sigma) at 4°C. After mechanical disruption, lysates were placed on ice for 15 minutes, and centrifuged at 14,000 rpm for 10 minutes at 4°C. The supernatants were collected and preserved at −70°C. Protein concentrations were determined by Coomassie Bradford Protein Assay Kit (Catalog No. 23200, Pierce, USA). The VEGF levels in the supernatant were determined by mouse VEGF ELISA kit (Quantikine; R&D Systems) at 450 nm to 570 nm, with an absorption spectrophotometer (ARVO™ MX 1420 multilabel counter, PerkinElmer, Kanagawa, Japan), and normalized to total protein, according to the manufacturer's protocols. Two eyes were needed to extract one protein sample, and eight mice in each group were examined.

### Western blot analysis of ERK phosphorylation

To determine whether INCB3344 treatment affected the MAPK signaling pathway in the laser-induced CNV model, the activation of ERK1/2 was assayed by ERK1/2 phosphorylation in the choroid-RPE complex using western blot analysis on Day 3. The protein extraction and the calculation of protein concentration were the same as the ELISA protocols. Eight µg of the total protein per sample was diluted with Laemmli Sample Buffer (Catalog No. 161-0737, Bio-Rad, CA), heated at 95°C for 5 min, separated by SDS-PAGE (Multigel?Mini, Cosmo Bio, Tokyo, JP), and electroblotted onto polyvinylidene fluoride membrane (PVDF, GE Healthcare, Buckinghamshire, UK). After blocking with 2.5% skim milk for 1 hour at room temperature, the membranes were incubated with a rabbit polyclonal anti-phospho-ERK antibody (1∶2000, Catalog No. 4370, Cell Signaling, Danvers, Massachusetts), a rabbit polyclonal anti-ERK to detect total ERK protein (1∶1000, Catalog No. 4695, Cell Signaling), or anti-GAPDH (14C10) (1∶1000, Catalog No. 2118, Cell Signaling) over night at 4°C. After washing with 0.1% Tris-buffered saline (TBS)-Tween, blots were incubated with horseradish peroxidase (HRP)-conjugated goat anti-rabbit IgG (1∶2500, Catalog No. 7074, Cell Signaling) for 1 h at room temperature. The blots were then washed three times with 0.1% TBS-Tween and the signals were visualized by an ECL kit (GE Healthcare, Buckinghamshire, UK) according to the manufacturer's protocol. The densities of immunoreactive bands were measured using Image J for Windows (NIH, Bethesda, Maryland). Eight mice in each group were examined.

### Effects on established CNV

We also evaluated the effect of INCB3344 on already established CNV as well as laser-induced, newly formed CNV. Spontaneous CNV was detected in 10–14 months old *Sod1* ^−/−^ mice by fundus photography and fluorescence angiography (FA) using a digital camera (CCD Color Video Camera, Sony, Japan) and recorded by IMAGEnet 2000 Digital Image System (Topcon, Japan). The fluorescein sodium solution (10%; 0.1 ml/kg; Fluorescite; Alcon, Fort Worth, TX) was injected into the intraperitoneal cavity of the mice. CNV was defined as present when early hyperfluorescence with late leakage was present at the site of dyed lesion during fundus examination. To evaluate drug treatment, 6 *Sod1* ^−/−^ mice with CNV received intravitreal injections of 1 µl INCB3344 (1800 nM), and 7 *Sod1* ^−/−^ mice with CNV received intravitreal injections of 1 µl vehicle. Fundus photography and FA were performed pre- and 1-week-post-treatment. Angiograms were obtained 5 min after the injection of fluorescein sodium solution, and the area of the CNV lesion was measured three times and averaged using Image J for Windows (NIH, Bethesda, Maryland).

### Statistical analysis

Results are expressed as the mean ± SE with *n* as indicated. Student's *t* test and one-way ANOVA was used for statistical comparison between two or three groups. Differences between the means were considered statistically significant at values of *P*<0.05.

## Results

### Histological evaluation and quantitative assessment of laser-induced CNV

**Figure 1:**
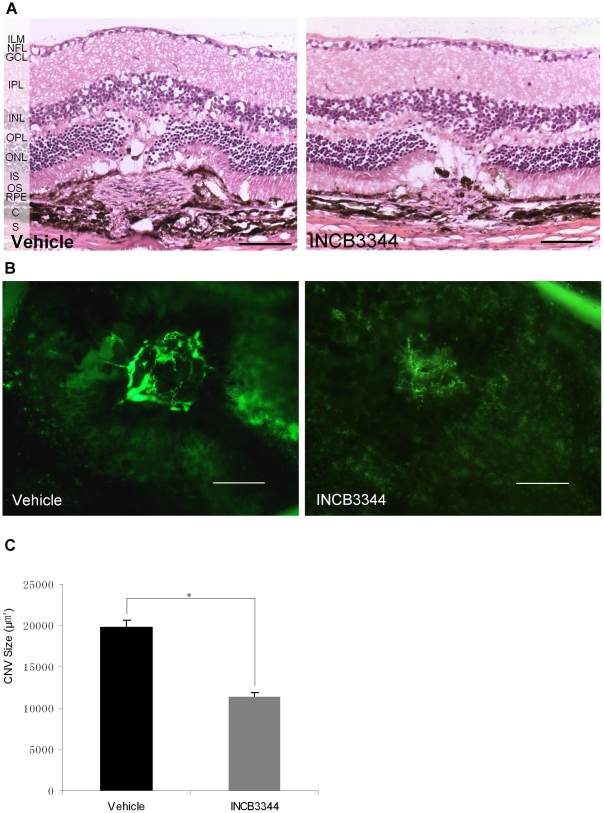
Effect of INCB3344 on CNV formation. (**A**) Haematoxylin–eosin-stained light micrograph of CNV lesions on Day 14 after laser photocoagulation. Each photograph shows the central area of CNV lesions in vehicle-treated or INCB3344-treated mice. Scale bar = 100 µm. (ILM: internal limiting membrane; NFL: nerve fiber layer; GCL: ganglion cell layer; IPL: inner plexiform layer; INL: inner nuclear layer; OPL: outer plexiform layer; ONL: outer nuclear layer; IS: inner segment; OS: outer segment; RPE: retinal pigment epithelium; C: choroid; S: sclera). (**B**) Representative micrographs of CNV lesions in the choroid-RPE flat mounts from laser-induced CNV in mice treated with vehicle or INCB3344. CNV areas were perfused with fluorescein isothiocyanate-dextran in flat-mount choroid-RPE complex. Scale bar = 100 µm. (**C**) Quantitative analysis of CNV size. Values are mean ± SE, vehicle, *n* = 78 spots, INCB3344, *n* = 81 spots. *P<0.001.

Histopathologic analysis showed that the CNV lesions in INCB3344-treated mice were smaller in diameter compared with those in vehicle-treated mice. Both groups had areas of fibro-vascular tissue comprising the vessel lumen, but the INCB3344-treated mice had smaller lesion areas comprising fibro-vascular tissue, disturbed RPE and pigment clumps compared with the vehicle-treated mice (Fig. 1A).

Choroid-RPE flat mounts confirmed the distinct reduction in CNV area by the INCB3344 treatment (Fig. 1 B). At Day 14 after photocoagulation, the mean CNV area was 19,759.5±861.1 µm^2^ in vehicle- treated mice (*n* = 78 spots), which significantly decreased in INCB3344-treated mice (11,392.2±468.8 µm^2^, *n* = 81 spots). This translated into a 42.4% decrease in CNV area by INCB3344 treatment (*P*<0.001) (Fig. 1C).

### Inhibition of macrophage infiltration by INCB3344 treatment

**Figure 2:**
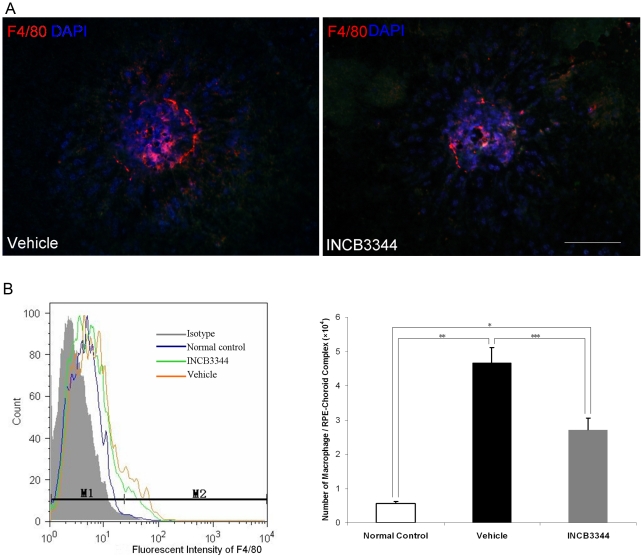
Macrophages detected by immunohistochemistry of choroid-RPE flat mounts and flow cytometry. (**A**) Immunohistochemistry of macrophages in choroid-RPE flat mounts on Day 3. After photocoagulation, a large number of macrophages accumulated at the laser injury sites. INCB3344 suppressed this increase. Scale bar = 100 µm. (**B**) Left: Overlay histogram of flow cytometric results. Right: Flow cytometric analysis data with F4/80 staining of the macrophages in choroid-RPE on Day 3 after laser photocoagulation (Macrophage numbers per choroid-RPE complex). After photocoagulation, the number of macrophages significantly increased compared with no laser photocoagulation controls (relative to normal control, ^*^ *P*<0.001 *n* = 5, ^**^ *P*<0.001 *n* = 5). INCB3344 treatment significantly reduced the number of macrophages compared to the vehicle-treated group (^***^ *P*<0.001, *n* = 5).

**Figure 3:**
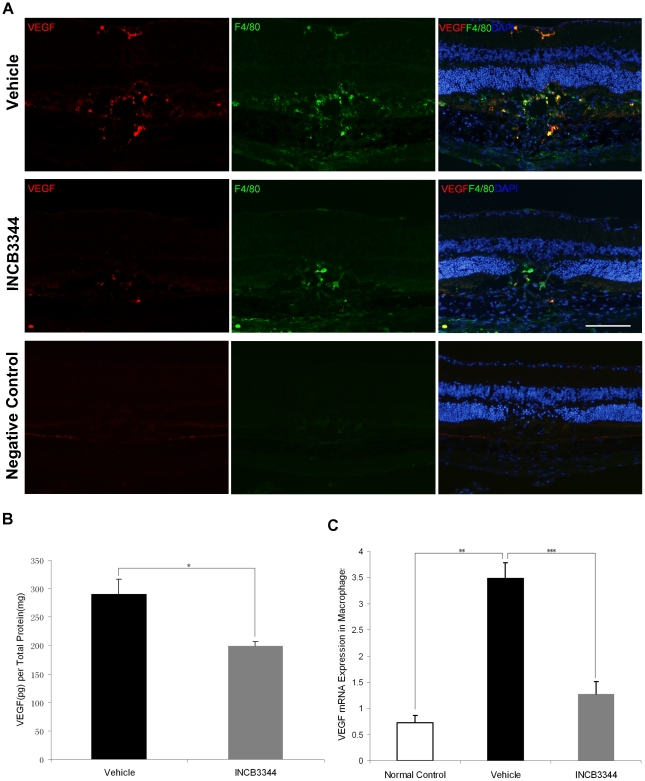
VEGF expression. (**A**) Immunohistochemistry of macrophages (green) and VEGF (red) in cryosections on Day 3. Significantly higher levels of VEGF were expressed in macrophages at the photocoagulated sites. VEGF localized mainly in infiltrating macrophages at the laser injury sites. INCB3344 apparently decreased VEGF immunoreactivity compared to vehicle treatment. The negative control omitting the primary antibody (second antibody only) had detectable auto-fluorescence in RPE. Scale bar = 100 µm. (**B**) VEGF protein levels in the choroid-RPE were quantitatively measured by ELISA. VEGF levels on Day 3 were significantly suppressed by INCB3344 treatment. (*n* = 8, *P = 0.012). (**C**) The expression of VEGF mRNA derived from macrophages isolated from choroid-RPE complexes was detected by real-time PCR on Day 3 after photocoagulation. The increased VEGF mRNA expression in infiltrating macrophages was significantly suppressed by INCB3344 treatment (**,***P<0.001, *n* = 3).

The number of F4/80 positive cells was substantially lower in INCB3344-treated mice than in vehicle-treated mice in both choroid- RPE flat mounts (Fig. 2A) and sections (Fig. 3A).

In the flow cytometry quantitative analyses, we detected only a few macrophages in normal mice (without laser treatment). On Day 3 after laser treatment, the number of macrophages dramatically increased in both vehicle- and INCB3344-treated mice, but INCB3344 treatment significantly suppressed the number of macrophages compared to vehicle treatment (Fig. 2B. *n* = 5, *P*<0.001).

### Decrease of VEGF expression by INCB3344 treatment

Strong VEGF-positive immunoreactivity was detected in the laser injury sites. The immunoreactivity was mainly localized to infiltrating macrophages (F4/80 positive cells) at the laser injury site. INCB3344 treatment decreased the VEGF immunostaining compared to vehicle treatment (Fig. 3A).

ELISA showed that VEGF protein levels in the choroid-RPE complex in INCB3344-treated mice were significantly lower (199.1±8.2 pg/mg) compared with vehicle-treated mice (289.3±27.8 pg/mg, *P* = 0.012, *n* = 8) (Fig. 3B).

Real-time PCR analysis on sorted macrophages from choroid-RPE complexes showed that VEGF mRNA expression significantly increased in those infiltrating macrophages after photocoagulation (*P*<0.001, *n* = 3), but it was markedly suppressed by INCB3344 treatment (*P*<0.001, *n* = 3) (Fig. 3C).

### Suppression of phosphorylated ERK1/2 (p-ERK1/2) by INCB3344 treatment

Phosphorylation of ERK1/2 is considered a measure of MAPK activation, which regulates a variety of angiogenic factors including VEGF. The inhibitory effects of INCB3344 on ERK1/2 phosphorylation in response to MCP-1 stimulation via CCR2 have been shown *in vitro* [26] [27]. Here, we further investigate the effect of INCB3344 on ERK1/2 phosphorylation in a CNV animal model.

**Figure 4:**
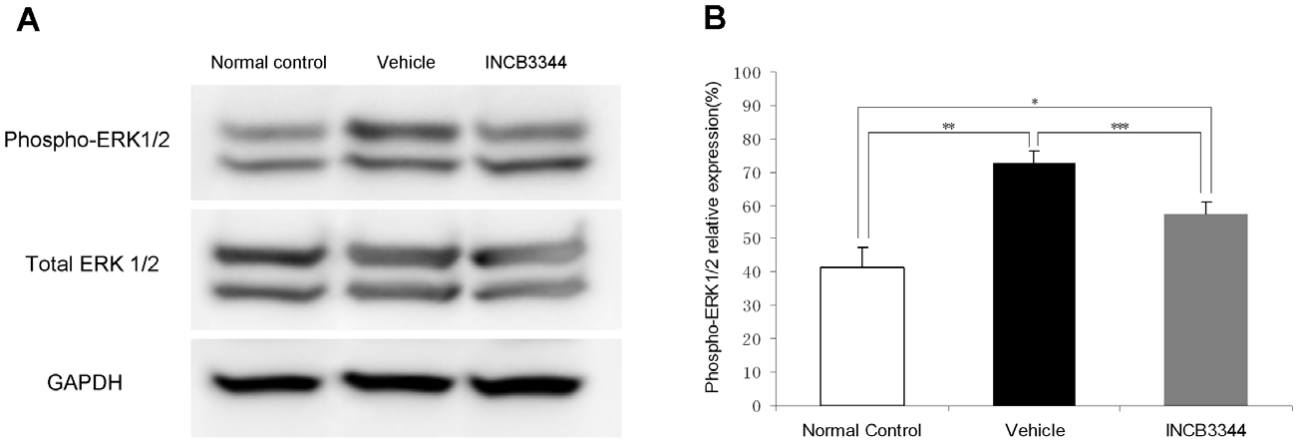
Phosphorylated extracellular signal-regulated kinase (p-ERK1/2) expression in Western blot. (**A**) A representative blot. p-ERK expression in the choroid-RPE complex from vehicle-treated mice and INCB3344-treated mice on Day 3 after laser photocoagulation, and normal mice (without photocoagulation). Western blot analysis revealed that p-ERK expression increased after laser photocoagulation and was suppressed by INCB3344 treatment. (**B**) Semi-quantitative analysis of the band intensity showed an increase in relative p-ERK expression (values normalized to total ERK expression) in the eyes of photocoagulated mice compared with untreated mice(*n* = 8, *P<0.001; *n* = 8, **P<0.001), and INCB3344 suppressed this increase (*n* = 8, ***P<0.001).

On Day 3 after photocoagulation, the p-ERK1/2 expression level increased relative to that of total ERK1/2 in the choroid-RPE complex in both vehicle- and INCB3344-treated mice compared with baseline levels (untreated normal mice), while the relative expression of p-ERK1/2 was significantly reduced in the INCB3344-treated mice compared with the vehicle-treated mice (*n* = 8, *P*<0.001) (Fig. 4).

### Regression of established CNV by INCB3344 treatment

**Figure 5:**
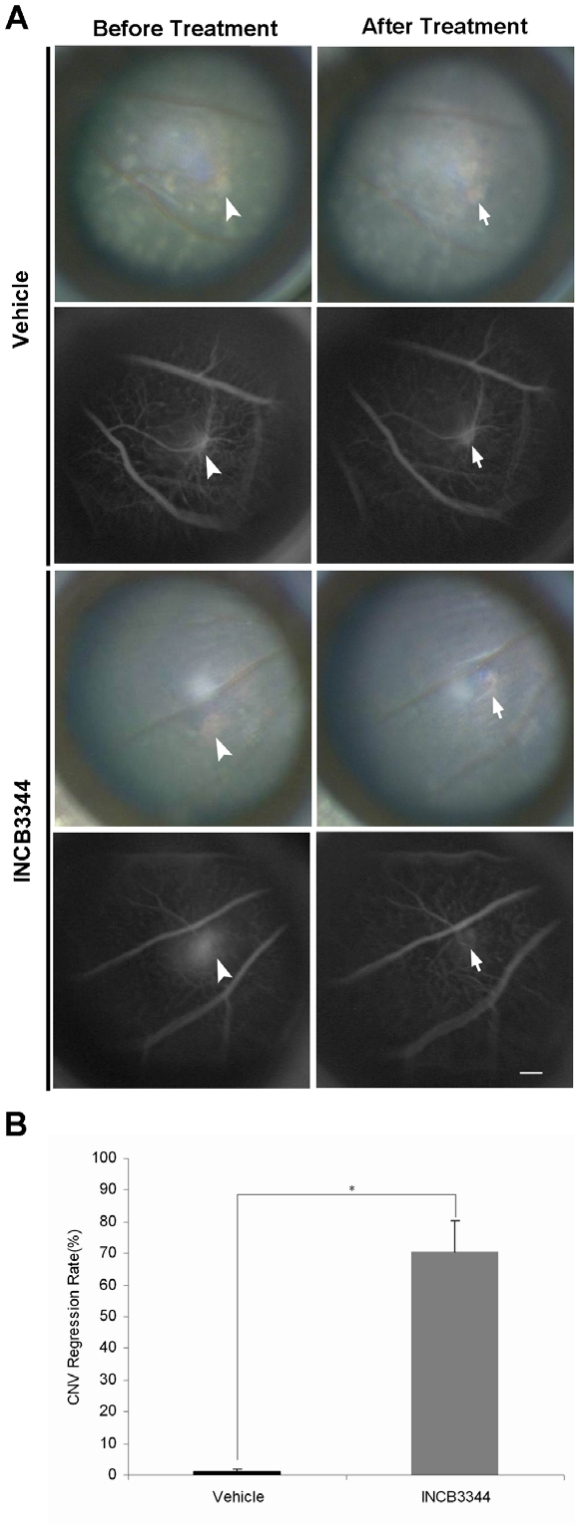
Effect of INCB3344 on established CNV. (**A**) Fundus photographs (1^st^ and 3^rd^ rows) and fluorescent angiography (2^nd^ and 4^th^ rows) pre-treatment (1^st^ column) and post-treatment (2^nd^ column) with vehicle (top 2 rows) or INCB3344 (bottom 2 rows). Established CNV in *Sod1* ^−/−^ mice were markedly regressed by INCB3344 treatment (bottom), while no significant regression of established CNV was detected in vehicle treatment (top). Scale bar = 20 µm. (**B**) INCB3344 treatment caused a 70.35±9.86% decrease (*n* = 6) in established CNV size, which was significantly higher than that in vehicle treatment (1.0±1.0%, *n* = 7, *P<0.001).

As previously reported, senescent *Sod1* ^−/−^ mice have many features in common with patients with AMD, such as sub-RPE deposits, thickened Bruch's membrane and CNV [32]. Spontaneous CNVs were detected in 13 *Sod1* ^−/−^ mice by both fundus examination and fluorescein angiography. INCB3344 treatment caused a regression in established CNV (Fig. 5A). The size of CNV significantly decreased from 2971.8±1976.3 µm^2^ (range from 172.4±9.2 µm^2^ to 12661.0±69.2 µm^2^, *n* = 6) before treatment to 1267.9±861.2 µm^2^ (range from 0 to 5452.3±74.4 µm^2^) at 1 week after treatment (*P*<0.001). Among them, two CNVs with the minimum sizes (172.4±9.2 µm^2^ and 219.7±9.8 µm^2^) were completely abolished by the INCB3344 treatment, which was confirmed by histological examinations in serial cryo-sections (Data not shown).

On the other hand, in vehicle-treated mice, no significant CNV regression was detected. CNV size was 1876.7±709.3 µm^2^ (range from 260.68±15.22 µm^2^ to 5466.4±6.44 µm^2^, *n* = 7) before treatment and 1872.2±707.6 µm^2^ (range from 249.60±7.19 µm^2^ to 5450.16±32.17 µm^2^) after treatment.

Paired pre-and post-comparison of CNV size showed that INCB3344 treatment caused a 70.35±9.86% decrease (*n* = 6) in CNV size (Fig. 5B), which was significantly higher than that in vehicle treatment (1±1%, *n* = 7, *P*<0.001).

## Discussion

In the present study, we evaluated the therapeutic value of INCB3344, a CCR2 antagonist, on a mouse model of age-related macular degeneration and demonstrated that INCB3344 treatment markedly suppressed new CNV formation and shrank established CNVs.

To investigate the possible cellular mechanism of INCB3344 suppression of CNV, we evaluated the infiltration of macrophages in the early phase of laser induced CNV and revealed that macrophage infiltration was significantly suppressed by INCB3344 treatment. In this study, we evaluated the macrophage infiltration on Day 3, the day of peak macrophage response [2], [13], [33]. We delivered INCB3344 to the vitreous cavity immediately after photocoagulation. After photocoagulation, local MCP-1 increases quickly [33], and recruits monocytes [34], [35] to laser injury sites, where they become inflammatory macrophages [36], [37]. Acting as a CCR2 antagonist, INCB3344 has displayed a high ability to inhibit monocyte chemotaxis in vitro and suppress macrophage influx in a variety of preclinical animal models of inflammatory diseases [26], [27]. Moreover, recent research reports that topical treatment of a CCR2 antagonist leads to the blocking of CCR2/MCP-1 interaction and reduces monocyte infiltration into the cornea in the dry eye disease mouse model [38]. These data are consistent with our results, which demonstrate that the suppression of monocyte/macrophage infiltration acts as an important cellular mechanism for INCB3344 treatment in the current model.

To determine the impact of INCB3344 on the downstream signaling of macrophages in CNV formation, we detected VEGF on the peak response day of macrophages. Our results demonstrated that VEGF significantly decreased with the suppression of infiltrating macrophages by INCB3344 treatment. After photocoagulation, VEGF is up-regulated [10], [11], [33] and acts as a promoting mediator in the development of CNV [11], [39], [40]. The variation of VEGF levels correlates highly to that of macrophages after laser injury [25], and their peak responses are reported to coincide with each other [13], [33]. While in pharmacologically macrophage-depleted mice [12], VEGF production is reduced in proportion to the decrease in the number of macrophages. Moreover, enriched ocular-infiltrating macrophages from laser-induced model mice have shown angiogenic ability in a dorsal air sac assay, and express activation-surface markers (class II, CD40, B7-1 and B7-2 molecules) and the mRNA for potential angiogenic factors including VEGF [16], which indicates that the infiltrating macrophages are a rich source of VEGF. Our results agree with these data and reveal that macrophages play an important role in the variation of intro-ocular VEGF after laser injury. Further more, our results demonstrate that elevated VEGF expression in infiltrating macrophages is suppressed by INCB3344. We reveal that INCB3344 can not only inhibit macrophage infiltration but also suppress the angiogenic ability of infiltrating macrophages, which results in the reduction of VEGF, and finally in suppression of CNV.

In this study, we revealed that INCB3344 treatment inhibited CNV formation via the suppression of macrophage infiltration. Our study focused on CCR2, macrophages, and VEGF, although several other cytokines such as tumor necrosis factor-alpha (TNF-α) [41]–[43], interleukin -1beta (IL-1β) [41], [43], hypoxia inducible factors (HIF-1α and HIF-2α) [44], IL-6 [35] and tissue factor (TF) [5] are reported to be involved in CNV formation. We cannot rule out a potential link between macrophages and other cytokines, however, VEGF is crucial in the pathogenesis of CNV formation because anti-VEGF drug therapy, for example, bevacizumab and ranibizumab have achieved an obvious effect in CNV due to age-related macular degeneration.

We demonstrated that the ERK1/2 phosphorylation induced by laser treatment was significantly suppressed by INCB3344. We examined the phosphorylation of ERK 1/2 because the activation of ERK1/2 in macrophages can be induced through the CCR2 [45] and their activation is thought to be a key component in the cellular events leading to the infiltration and activation of macrophages [46]–[48]. Our results are consistent with the results *in vitro* [20], [21] and the previous reports that blockage of CCR2 by anti-CCR2 monoclonal antibodies inhibits phosphorylation of ERK1/2 in peritoneal macrophages [45]. Our results on the inhibitory effect on VEGF secretion are also compatible with the data that ERK1/2 activation in macrophages [49] or monocytes [34] is reported to be responsible for VEGF production in these cells. We can conclude that INCB3344 inhibits the activation of ERK1/2 in macrophages by blocking CCR2, which results in the reduction of macrophage infiltration and VEGF production.

We further evaluated the therapeutic effect of INCB3344 on established CNV in *Sod1* ^−/−^ mice. SOD1 is a component of the antioxidant defense system of the retina, and it has been demonstrated that SOD1 deficiency leads to retinal dysfunction and progressive, degenerative changes of retinal cell layers [50]. Senescent *Sod1* ^−/−^ mice have many features in common with patients with AMD, such as age-related accumulation of sub-RPE deposits, thickened Bruch's membrane, and spontaneous CNV, which recapitulates the key elements of the human pathology [32]. In this study, we detected the established spontaneous CNV by screening senescent *Sod1* ^−/−^ mice, and then investigated the therapeutic effect of INCB3344 on this CNV model. This process can closely mimic the diagnosis and treatment of an AMD patient with CNV. Although the size of these CNV varied in the *Sod1* ^−/−^ mice, by paired pre- and post-comparison, we demonstrated that INCB3344 treatment caused marked regression of established CNV. In our results, fluorescence leakage in CNV area significantly reduced or disappeared in FA by the treatment of INCB3344, whereas the lesion area did not show much amelioration in fundus photographs. This discrepancy may be due to the accumulation of irreversible histological damage in the CNV area including sub-RPE deposits or fibrosis, and future investigations with more samples for longer duration, and evaluation with optical coherence tomography may be helpful.

In this study, we showed that blockage of the CCR2/MCP-1(CCL2) signal pathway by INCB3344 suppressed the development of CNV in laser induced model. However, there is some controversy over the issue of CNV formation associated with the CCR2/MCP-1 signal pathway. Ambati et al. [51] detected spontaneous CNV in senescent Ccl-2 (MCP-1) KO mice and Ccr-2 KO mice, while Luhmann et al. [30] were unable to detect any spontaneous CNV in similarly aged Ccl-2 KO mice and revealed that the AMD-like features in MCP-1 KO mice described by Ambati et al. might be the result of aging alone. Although these discrepancies need further investigation, the size of laser induced CNV was reduced in MCP-1 KO mice [30] and Ccr-2 KO mice [16] with fewer macrophages compared with wild type mice. The results of these reports are consistent with our conclusion that suppression of macrophage migration via blockage of CCL-2/CCR-2 with INCB3344 inhibits CNV formation in a laser-induced CNV model.

In summary, we demonstrated that local administration of the CCR2 antagonist, INCB3344, effectively inhibited laser-induced CNV formation in mice through the suppression of macrophage infiltration and VEGF expression of infiltrating macrophages. In addition to this preventive effect, INCB3344 caused marked regression of established CNV. Therefore, we propose that INCB3344 may act as an attractive therapeutic approach for the treatment of CNV in AMD.
